# PCSK9 is minimally associated with HDL but impairs the anti-atherosclerotic HDL effects on endothelial cell activation

**DOI:** 10.1016/j.jlr.2022.100272

**Published:** 2022-09-05

**Authors:** Ioannis Dafnis, Aikaterini N. Tsouka, Christina Gkolfinopoulou, Constantinos C. Tellis, Angeliki Chroni, Alexandros D. Tselepis

**Affiliations:** 1 Institute of Biosciences and Applications, National Center for Scientific Research "Demokritos", Agia Paraskevi, Athens, Greece; 2 Atherothrombosis Research Centre, Department of Chemistry, University of Ioannina, Ioannina, Greece

**Keywords:** ApoA-I, Atheroprotection, Cardiovascular disease, LDL, Lp(a), plasma, apoB, PCSK9 inhibition, ROS, furin, DPBS, Dulbecco's phosphate-buffered saline, I-HDL, Intermediate-HDL, L-HDL, Large-HDL, PCSK9, Proprotein Convertase Subtilisin/Kexin type 9, rHDL, reconstituted HDL, ROS, reactive-oxygen species, S-HDL, Small-HDL

## Abstract

Proprotein Convertase Subtilisin/Kexin type 9 (PCSK9) regulates the cell-surface localization of LDL receptors in hepatocytes and is associated with LDL and lipoprotein(a) [Lp(a)] uptake, reducing blood concentrations. However, the connection between PCSK9 and HDL is unclear. Here, we investigated the association of plasma PCSK9 with HDL subpopulations and examined the effects of PCSK9 on the atheroprotective function of HDL. We examined the association of PCSK9 with HDL in apoB-depleted plasma by ELISA, native PAGE, and immunoblotting. Our analyses showed that upon apoB-depletion, total circulating PCSK9 levels were 32% of those observed in normolipidemic plasma, and only 6% of PCSK9 in the apoB-depleted plasma, including both the mature and furin-cleaved forms, was associated with HDL. We also show human recombinant PCSK9 abolished the capacity of reconstituted HDL to reduce the formation of ROS in endothelial cells, while a PCSK9-blocking antibody enhanced the capacity of human HDL (in apoB-depleted plasma) to reduce ROS formation in endothelial cells and promote endothelial cell migration. Overall, our findings suggest that PCSK9 is only minimally associated with HDL particles, but PCSK9 in apoB-depleted plasma can affect the atheroprotective properties of HDL related to preservation of endothelial function. This study contributes to the elucidation of the pathophysiological role of plasma PCSK9 and highlights further the anti-atherosclerotic effect of PCSK9 inhibition.

Proprotein Convertase Subtilisin/Kexin type 9 (PCSK9) is a serine protease that plays a major role in the pathophysiology of atherosclerosis and is significantly associated with an increased risk of cardiovascular events (1). Increased plasma levels of PCSK9 have been reported in patients with acute coronary syndromes (2). PCSK9 has been identified as a central regulator of plasma LDL-cholesterol levels through its ability to bind to LDL-Receptor (LDLR) and mediate LDLR degradation in the liver (3). The binding of PCSK9 on LDLR inhibits the release of LDLR from the LDLR/LDL complex in the early endosomes and directs the receptor for degradation in late endosomes/lysosomes, thus preventing the recycling of LDLR to the plasma membrane (4, 5). Cholesterol-lowering treatments with statins or ezetimibe have been shown to increase circulating PCSK9 levels in humans (6, 7, 8), which may limit their efficacy at lowering plasma LDL-cholesterol levels. Inhibition of PCSK9 has been shown to reduce risk of cardiovascular events in patients with atherosclerotic cardiovascular disease (9). PCSK9 inhibitors Alirocumab and Evolocumab, two human mAbs, are currently used in the clinical practice and can decrease LDL-cholesterol levels up to 73%, while they also reduce the risk of atherosclerotic cardiovascular disease (10).

PCSK9 is mainly expressed by the liver (11) and secreted into the circulation. PCSK9 circulates in plasma in different molecular forms that include a mature form, a furin-cleaved form, and high molecular weight self-associated forms (12, 13). The mature form of PCSK9 is a heterodimer of the autocatalytically cleaved proprotein and prodomain (∼ 62+13 kDa) (14, 15), and the furin-cleaved form is a 55 kDa protein produced by proteolysis at the amino-terminal site of mature form by the protease furin (14, 15). The furin-cleaved PCSK9 form circulates in blood at lower concentrations and is less active in the degradation of LDLR and removal from cell surface, compared with the mature PCSK9 (14, 16, 17, 18). The pathophysiological role of the different PCSK9 forms in plasma remains unclear.

Plasma PCSK9 levels correlate positively with plasma LDL-cholesterol and Lpa levels (19). It has been shown that a substantial fraction of mature PCSK9 is associated with LDL and Lp(a) particles, while most of the furin-cleaved form can be found in the apoB-free fraction of plasma (13, 20, 21) that contains the HDL particles. Regarding the correlation of PCSK9 with HDL-cholesterol in human plasma, some studies have demonstrated a positive correlation between HDL-cholesterol and PCSK9 levels (22, 23) whereas others failed to show this correlation (24, 25). In addition, there are contrasting results about the association of human PCSK9 with HDL particles (26). Α study failed to show any association (27), whereas others demonstrated that PCSK9 binds HDL in the circulation (20, 26). Interestingly, a recent study suggests that the main carrier of PCSK9 in plasma are the HDL particles (26) that contain both mature and furin-cleaved PCSK9 forms (26).

Contrasting results concerning the association of PCSK9 with HDL have also been obtained in human PCSK9 transgenic mice. Western blot analysis of lipoprotein fractions isolated from serum of PCSK9 transgenic mouse by FPLC showed the presence of hPCSK9 in HDL and lipoprotein-free fractions (12). In another study though, there was no detection of apoA-I via immunoblot following immunoprecipitation with anti-PCSK9 antibodies of hPCSK9 transgenic mouse serum (20).

HDL has several atheroprotective properties as it performs the reverse cholesterol transport and exhibits anti-oxidative, anti-inflammatory, anti-thrombotic, and endothelial-protective properties (28). The most studied antiatherogenic function of HDL is its capacity to participate in the reverse cholesterol transport (29). In this regard, a previous study has suggested that PCSK9 inhibits the ABCA1-dependent cholesterol efflux from macrophages in the presence of apoA-I as acceptor (30). However, it remains to be established whether PCSK9 contributes to other atheroprotective functions of HDL, such as the induction of endothelial cell migration, an important event for the regeneration of the dysfunctional or damaged artery wall endothelium, as well as the reduction of oxidative stress. Both endothelial damage or dysfunction and oxidative stress play a major role in atherogenesis (31).

Therefore, the aim of the present study was to investigate the following: i) the possible association of PCSK9 with HDL and ii) the role of HDL-associated PCSK9 on the capability of this lipoprotein to induce endothelial cell migration and to reduce reactive-oxygen species (ROS) formation.

## Materials and methods

### Preparation of apoB-depleted plasma

Peripheral venous blood samples were collected from apparently healthy normolipidemic volunteers (6 men, aged 30 ± 13.9, LDL-cholesterol ≤ 105 mg/dl, triglycerides ≤98 mg/dl, HDL-cholesterol ≥ 48 mg/dl, without receiving any hypolipidemic treatment) after 8–12 h of fasting, into vacutainer containing EDTA. Plasma was separated by low-speed centrifugation (1500 *g* ×15 min) and subsequently incubated for 10 min at room temperature with a solution consisted of 10 g/L dextran sulfate and 0.5 M MgCl_2_._2_O, pH 7.0, at a volume ratio of 10:1, respectively to precipitate all apoB-containing lipoproteins. The supernatant (apoB-depleted plasma) was separated from the precipitant by centrifugation at 1,500 *g* for 10 min (32, 33). The HDL-cholesterol levels in apoB-depleted plasma were determined by commercially available enzymatic colorimetric method following the manufacturer’s instruction using the Bio-Merieux reagents as we have previously described (34, 35). The lipoprotein content of apoB-depleted plasma was verified by agarose gel electrophoresis using the lipoprotein separation kit Hydragel LIPO+Lp(a) K20 (Sebia), following the manufacturer’s instructions. Briefly, 10 μl of apoB-depleted plasma were loaded on the gel and run for 90 min at 50 V. The gel was dried and stained with Sudan black for identification of lipoprotein classes (36, 37).

### HDL isolation by ultracentrifugation

HDL was isolated from apoB-depleted plasma by potassium bromide flotation ultracentrifugation at d=1.063–1.210 g/ml in a Beckman L70 ultracentrifuge at 40,000 rpm, 14°C, for 1 with a type NVT65 rotor as we have previously described (33). The HDL-containing lipoprotein fraction recovered on the top of the tube was dialyzed against 10 mM PBS (pH 7.4) for 24 h at 4°C. It was then filter sterilized and stored at 4°C in the dark under nitrogen for up to 2 weeks. The HDL-cholesterol content was determined by the same enzymatic colorimetric method described above.

### HDL subfractionation by nondenaturing gradient polyacrylamide gel electrophoresis

HDL particles were separated by nondenaturing high resolution 4%–30% gradient PAGE, using the Lipoprint System HDL subfraction Kit (Quantimetrix, Redondo Beach, CA) as we have previously described with some modifications (38). Samples from total plasma (50 μl) were placed on the upper part of the tube containing the 4%–30% gradient polyacrylamide gel and then mixed with 300 μl of Lipoprint Loading Gel solution. It contains the lipophilic dye Sudan Black, which stains all lipids (39). After 30 min of photopolymerization at room temperature, electrophoresis was performed for 50 min with 3 mA for each gel tube. Ten HDL fractions were separated: fractions 1–3 were denoted as Large-HDL (L-HDL), fractions 4–7 constituted the Intermediate-HDL (I-HDL), and fractions 8–10 were denoted as Small-HDL (S-HDL) subfraction (38). To determine the PCSK9 on each HDL subfraction, two runs were simultaneously performed for each plasma sample and the bands corresponding to L-HDL, I-HDL, and S-HDL were excised from the 2 different gels, jointed, and minced into small pieces, which were stored in Eppendorf tubes at −20°C. The frozen gels were crushed with a rod and each HDL subfraction was then extracted by incubation of the gel with 150 mM Tris buffer, pH 6.8, containing 1% SDS (v/v) for 30 min, followed by centrifugation at 13,000 rpm for 5 min at room temperature. The supernatant was collected, and the pellet was again submitted to the above extraction procedure. The joint supernatants from each tube were then concentrated using Amicon ultracentrifugal filter (Merck) and analyzed for their PCSK9 content as described below. The yield of the method was calculated by the recovery of PCSK9 using a known concentration of PCSK9. In brief, 25 μg of recPCSK9 were added in the gel which were extracted as described above. The yield of the method ranged from 50% to 60%. In parallel, a gel tube not containing any plasma was treated as above and the total region on which the HDL subfractions migrate was excised and then we performed PCSK9 determination as described below. Moreover, the same gel tube before excision was run using the Lipoprint assay.

### Measurement of PCSK9 levels

The concentration of PCSK9 in total plasma, apoB-depleted plasma, HDL isolated by ultracentrifugation, and the three HDL subfractions, separated by nondenaturing PAGE using the Lipoprint System, was determined by a quantitative sandwich enzyme immunoassay method (R&D Systems) in which a monoclonal antibody specific for the human PCSK9 was used, following the manufacturer instructions, as we have previously described (40). In an effort to investigate whether the PCSK9 levels determined with this immunoassay are affected in the presence of HDL, we performed preliminary experiments in which we determined the PCSK9 levels in three PCSK9 standard solutions in the calibrator diluent of the ELISA kit (concentrations 2.5, 10, and 20 ng/ml) in the presence or absence of HDL isolated by ultracentrifugation. The color intensity in each well was measured at 450 nm, using a microplate reader (Tecan infinite 200 PRO). In all measurements, the PCSK9 concentration was expressed in ng/ml. The intra-assay coefficient of variation ranges from 4.1 to 6.5, and the inter-assay coefficient of variation ranges from 4.1 to 6.0.

### Measurement of HDL mass

The mass of HDL was determined by a commercially available ELISA method (Aviva Systems Biology OKEH00700), following the manufacturer instructions. The color intensity in each well was measured at 450 nm, using a microplate reader (Tecan infinite 200 PRO). The intra- and inter-assay coefficients of variation are ≤ 4.3% and ≤ 7.5%, respectively. The HDL mass was determined in the apoB-depleted plasma as well as in the aspirated supernatants of the HDL Elisa microplate, after the incubation of the ApoB-depleted plasma samples in the plates of the HDL ELISA Kit, as described in the next paragraph.

### Determination of PCSK9 associated with HDL in apoB-depleted plasma

To determine the PCSK9 content of HDL in apoB-depleted plasma, we combined two different ELISA kits, the HDL ELISA Kit (Aviva Systems OKEH00700), and the ELISA kit for PCSK9 measurement, described above. ApoB-depleted plasma samples (150 μl from each sample containing 16.7 ± 5.2 ng PCSK9) were added on each well of the HDL Elisa microplate, containing the antibody against HDL, and incubated for 2 h at 37°C to allow the HDL binding. The supernatant was subsequently removed by aspiration and submitted to analysis for its cholesterol content using the enzymatic method described above as well as for its HDL content using HDL ELISA Kit. The plate was then washed with wash buffer (1×) and subsequently incubated for at 25°C with the horseradish peroxidase-conjugated anti-human PCSK9 antibody from the PCSK9 ELISA Kit. The plate was then washed and the PCSK9 levels (in ng) were measured using the PCSK9 ELISA Kit. The assay coefficient of variation is 3.2%.

### Western blot analysis

Western blot analysis of PCSK9 was performed using a sheep polyclonal anti-human PCSK9 antibody (R&D systems), diluted 1:1.000 (v/v), as the primary antibody, and a donkey horseradish peroxidase-coupled anti-sheep IgG (Agrisera Antibodies), diluted 1:2.500 (v/v), as the secondary antibody.

### Determination of ApoA-I

ApoA-I concentrations in human apoB-depleted plasma were determined using the commercially available reagents for quantitative immunoturbidimetric determination of ApoA-I (Sentinel Diagnostics).

### Preparation of reconstituted HDL particles

Reconstituted HDL (rHDL) particles containing apoA-I, phosphatidylcholine, and cholesterol were prepared using 1-palmitoyl-2-oleoyl-sn-glycero-3-phosphocholine (Sigma-Aldrich, Germany), cholesterol (Sigma-Aldrich), recombinant human apoA-I (produced as described (41)), and sodium cholate in a molar ratio 100:10:1:100 in 10 mM Tris-HCl buffer, pH 8, containing 0.01% EDTA, 150 mM NaCl (41).

### Measurement of ROS generation

Intracellular ROS generation in EA.hy926 cells was measured as described previously (42). Briefly, EA.hy926 cells, cultured in DMEM supplemented with 10% FBS and antibiotics, were plated in 12-well plates at a density of 1.8 × 10^5^ cells/well. When cells reached 90% confluency, they were synchronized in DMEM/antibiotics medium for 16 h and then were incubated with DMEM in the presence or absence of 1 μM (at apoA-I concentration) rHDL particles or 0.16 μM (at apoA-I concentration) human HDL (apoB-depleted plasma) for 24 h at 37 °C. In addition, cells were incubated, for 24 h at 37°C, with DMEM in the presence of 1 μM rHDL that had been preincubated with 2 μg/ml recombinant human PCSK9 (Cayman Chemical) for 30 min at 37°C or 0.16 μM human HDL (apoB-depleted plasma) preincubated with 2.5 μg/ml blocking antibody for PCSK9 (sheep polyclonal anti-human PCSK9 antibody, R&D Systems) for 1 h at 37°C or 0.16 μM human HDL (apoB-depleted plasma) preincubated with 2.5 μg/ml of normal isotype IgG (2A3, BioXCell) for 1 h at 37°C. The concentration of human recombinant PCSK9 has been selected based on a previous study (43). After incubation, cells were washed with DMEM and further incubated in the dark for 45 min at 37 °C in DMEM containing 25 μΜ 2′,7′-dichlorodihydrofluorescein diacetate (Molecular Probes/Thermo Fisher Scientific). Subsequently, cells were washed with Dulbecco's phosphate-buffered saline (DPBS) and production of ROS was detected by recording the fluorescence of 2′,7′-dichlorofluorescein with a Zeiss Axiovert 25 Inverted microscope equipped for fluorescence microscopy (excitation 450–490 nm, emission 520 nm). 2′,7′-dichlorofluorescein fluorescent intensity was measured for at least 30 cells from the fluorescent images of each sample using the ImageJ analysis software and the relative fluorescence intensity was taken as average of values of at least five images for each experiment.

### Endothelial cell migration assay

For endothelial cell migration analysis (44, 45), EA.hy926 cells were plated in 24-well plates at a density of 8 × 10^4^ cells/well. When cells reached 90% confluency, they were synchronized in DMEM/antibiotics medium for 16 h and then cell monolayers were scratched using a pipette tip (200 μl). Wounded cells were rinsed with warm DPBS to remove nonadherent cells and then EA.hy926 cells were incubated with 0.35 μM (at apoA-I concentration) human HDL (apoB-depleted plasma), for 1 h at 37°C with or without 2.5 μg/ml of blocking antibody for PCSK9, in DMEM/antibiotics medium for 24 h. At the end of the incubation period, cells were fixed with ice-cold methanol for 15 min, permeabilized in 0.1% Triton X-100/DPBS for 15 min, and stained with hematoxylin (Sigma Aldrich) for 5 min. Cells were photographed on marked positions at 0 and 24 h, using the 10x objective lens of a Zeiss Primovert Inverted microscope (Zeiss, Germany). Wound healing was quantified, following analysis of images with ImageJ analysis software (NIH, Bethesda, MD) (46), by measuring the number of cells that migrated.

### Statistical analysis

Quantitative data are presented as means ± SD. Statistical analyses were conducted using the GraphPad Prism™ software.

## Results

### Association of PCSK9 with HDL

**Fig. 1:**
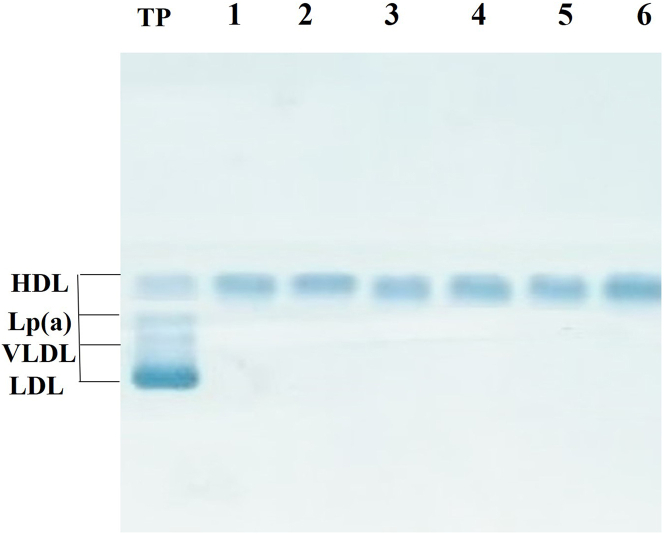
Agarose gel electrophoresis of plasma and apoB-depleted plasma samples. Lanes 1–6: Lipoprotein profiles of apoB-depleted plasma samples from 6 different donors, which contain only HDL. Electrophoresis was performed on buffered (pH 8.5) agarose gels using the semi-automated HYDRASYS instrument. Ten microliters of total or apoB-depleted plasma were loaded on the gel and run for 90 min at 50 V. The gel was dried and stained with Sudan black for identification of lipoprotein classes. The excess of stain was removed with an alcoholic solution. TP: Typical lipoprotein profile of total plasma.

To study the possible association of PCSK9 with HDL in human plasma, we prepared apoB-depleted plasma samples from six different normolipidemic volunteers, following the procedure described in “Materials and methods” section. This method precipitates all apoB-containing lipoproteins, thus allowing the separation of HDL (that remains in the plasma supernatant) from these lipoproteins, under milder conditions as compared to ultracentrifugation methods which use high g forces, and have been shown to affect the proteomic composition of lipoproteins, including HDL (47). Under these conditions, a dissociation of PCSK9 from HDL particles has been reported (48). The lack of apoB-lipoprotein particles in HDL-containing plasma supernatants (apoB-depleted plasma) was verified by agarose gel electrophoresis (Fig. 1). The HDL-cholesterol levels in these supernatants were 44.5 ± 21.5 mg/dl. The PCSK9 levels in total plasma were 344 ± 94 ng/ml, whereas the levels in apoB-depleted plasma were 111 ± 35 ng/ml. This indicates that the 32.3 ± 10.3% of total PCSK9 in normolipidemic plasma is not associated with apoB-lipoproteins and remains in the supernatant after precipitation of these lipoproteins. It should be noted that the solution used to precipitate the apoB-lipoproteins does not affect the PCSK9 quantitation assay, as it was demonstrated in preliminary experiments, in which we determined the PCSK9 levels using the recombinant human PCSK9 standard, provided by the PCSK9 ELISA Kit (R&D Systems), before and after the addition of this solution.

Since the apoB-depleted plasma contains both, HDL and all plasma proteins, we next evaluated whether all the remaining PCSK9 in apoB-depleted plasma is associated with HDL. In this regard, we used a combination of two different ELISA kits, the HDL ELISA Kit and the ELISA kit used for PCSK9 quantitation, following the procedure described in “Materials and methods”. To ensure that all HDL particles of each apoB-depleted plasma sample were captured in each well of the plate from the HDL ELISA Kit, we determined the cholesterol levels in the aspirated supernatants, after a incubation of the apoB-depleted plasma samples in the plate, as we described in “Materials and methods”. There were no detectable cholesterol levels in these supernatants, indicating that all HDL particles were captured in each well of the microelisa plate. To further investigate whether all HDL was captured in each well, we determined the HDL levels in the aspirated supernatants (using the HDL ELISA Kit). There were no detectable HDL levels in these supernatants thus further showing that HDL was quantitatively captured in the wells.

The PCSK9 levels determined in the wells containing the captured HDL (HDL-associated PCSK9) were 1.03 ± 0.11 ng, which corresponds to the 6.18 ± 2.11% of the total PCSK9 found in apoB-depleted plasma. This indicates that the majority of the PCSK9 found in the apoB-depleted plasma is not associated with HDL but exists in a free protein form. To investigate whether ultracentrifugation could affect the PCSK9 levels on HDL, we submitted the apoB-depleted plasma into ultracentrifugation to prepare HDL and then determined the PCSK9 levels and cholesterol levels on the isolated HDL particles. According to our results, the HDL preparations contained 0.041 ± 0.009 ng PCSK9 / mg HDL-cholesterol, which was significantly lower to that determined in the apoB-depleted plasma (249.4 ± 162.7 ng PCSK9 / mg HDL-cholesterol), indicating that ultracentrifugation significantly reduces the PCSK9 content of HDL.

**Fig. 2:**
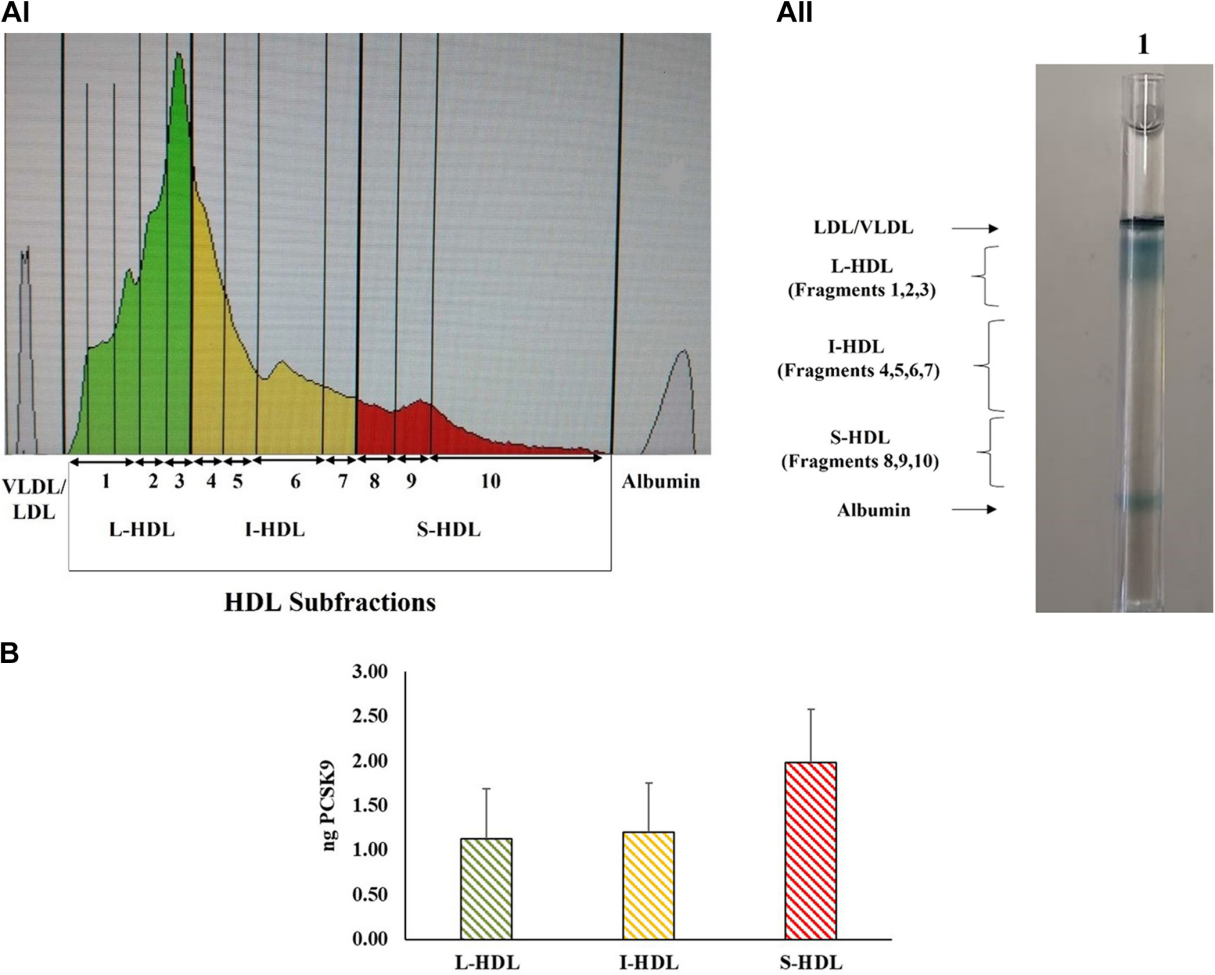
PCSK9 levels in HDL subfractions separated in nondenaturing gradient polyacrylamide gel tubes using the Lipoprint System. AI: Representative profile of HDL subfractions. Distribution of HDL subfractions, Large-HDL (L-HDL), Intermediate-HDL (I-HDL), and Small-HDL (S-HDL). Subfractionation of HDL from six normolipidemic healthy volunteers was performed by the Lipoprint System HDL subfraction kit. AII: Representative profile of HDL fractions in the gel tube after total plasma electrophoresis. B: Amount of PCSK9 in each HDL subfraction expressed as ng. The bands corresponding to L-HDL, I-HDL, and S-HDL were excised from two different gels ran in parallel, jointed, and minced into small pieces. Each HDL subfraction was then extracted from the gel by incubation with 150 mM Tris buffer, pH 6.8, containing 1% SDS (v/v) for 30 min, followed by centrifugation at 13,000 rpm for 5 min at room temperature. Each supernatant was concentrated using Amicon ultra centrifugal filter (Merk USA) and submitted to analysis of its PCSK9 content. PCSK9, Proprotein Convertase Subtilisin/Kexin type 9.

We further studied the distribution of PCSK9 among HDL subfractions, separated by nondenaturing 4%–30% gradient PAGE using total plasma samples, as described in “Materials and methods”. The distribution of the three HDL subfractions L-HDL, I-HDL, and S-HDL, which correspond to the 10 HDL fractions separated by this method, as well as a representative electrophoresis tube showing the stained HDL bands, are demonstrated inFig. 2AI, AII, respectively. The parts of the gel corresponding to L-HDL, I-HDL, and S-HDL were excised and proceed for the determination of PCSK9 content as described in “Materials and methods”. Figure 2B demonstrates the levels of PCSK9 migrated in the area of each HDL subfraction. These results further support the association of PCSK9 with HDL and show that it is preferentially associated with the small S-HDL particles. Furthermore, to determine whether the gel itself could have produced false-positive signals of PCSK9, the total region on which HDL subfractions migrate from a gel tube not containing plasma was submitted to PCSK9 determination, as described in “Materials and methods”. The absorbances of this region with the ELISA kit for PCSK9 determination were 0.038 ± 0.013 at the level of blank wells, which contain the calibrator diluent according to manufacturer’s instructions, indicating that there is no false-positive signal. Moreover, as concerned of the HDL levels, the Lipoprint assay itself provides that the absorbance of the gel tube not containing plasma is at the levels of zero. Therefore, even for HDL, there is no any false-positive signal.

**Fig. 3:**
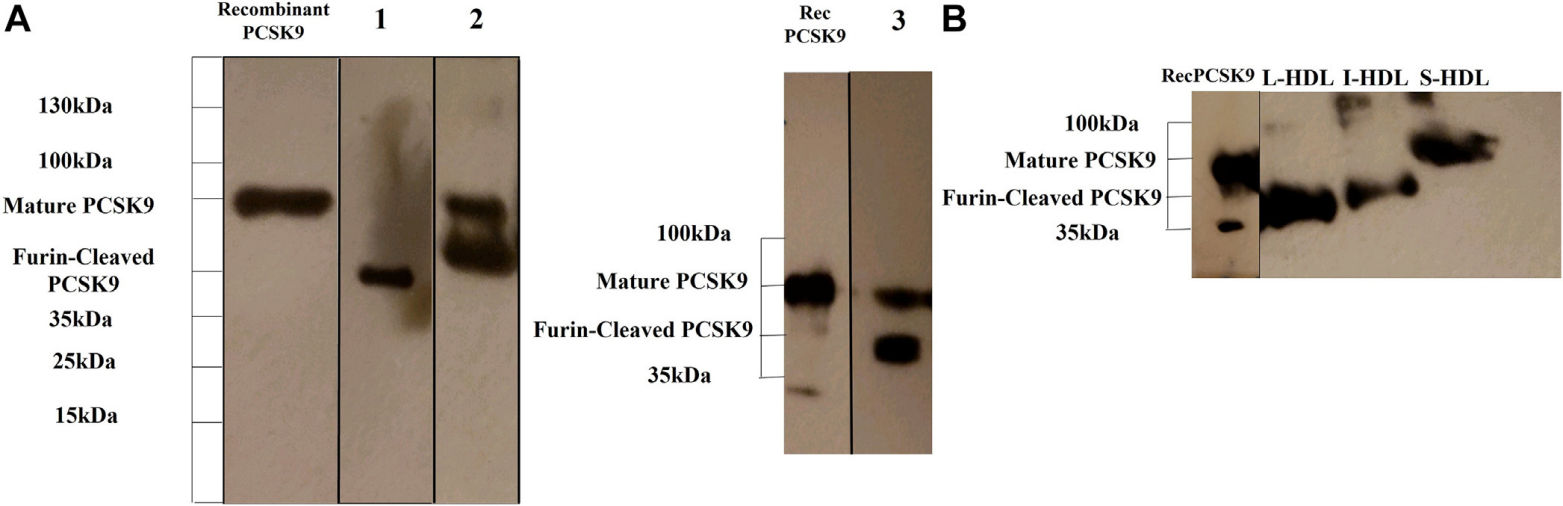
PCSK9 determination by Western Blot analysis of PCSK9 in ApoB-depleted plasma and in HDL subfractions. A1: HDL isolated from apoB-depleted plasma by ultracentrifugation. A2: ApoB-containing precipitant prepared by centrifugation of plasma treated with Dextran sulfate/MgCl_2_ at 1,500 *g* for 10 min. The precipitant solubilized with SDS, before electrophoresis. A3: ApoB-depleted plasma (the supernatant from plasma treated with Dextran sulfate/MgCl_2_ to precipitate all ApoB-containing lipoproteins, followed by centrifugation at 1,500 *g* for 10 min to remove the precipitant). B: HDL subfractions separated by the Lipoprint system, 20 μg of total protein of L-HDL and I-HDL and 10 μg of total protein of S-HDL were loaded. I-HDL, Intermediate-HDL; L-HDL, Large-HDL; PCSK9, Proprotein Convertase Subtilisin/Kexin type 9; S-HDL, Small-HDL.

We next performed Western Blot analysis after SDS-PAGE to determine the form of PCSK9 (mature or furin-cleaved) associated with HDL. As it is shown in Fig. 3A3, both PCSK9 forms exist in the apoB-depleted plasma, which contains the HDL-associated and free PCSK9. Both PCSK9 forms were also detected in the apoB-precipitant after treatment of total plasma with dextran-Mg^2+^ (Fig. 3A2). By contrast, HDL isolated by ultracentrifugation of apoB-depleted plasma contains only the furin-cleaved PCSK9 form (Fig. 3A1). This suggests that either only this form is associated with HDL or ultracentrifugation leads to a complete dissociation of the mature PCSK9 from HDL particles. In support of the later suggestion, Western blot analysis of the three HDL subfractions obtained from the nondenaturing PAGE of total plasma samples showed that L-HDL and I-HDL contain only furin-cleaved PCSK9, whereas S-HDL contain only the mature PCSK9 form (Fig. 3B). This suggests that total HDL contain both forms of PCSK9, however, they are differentially distributed among the HDL subfractions.

### Effect of PCSK9 on endothelial-protective effects of HDL

**Fig. 4:**
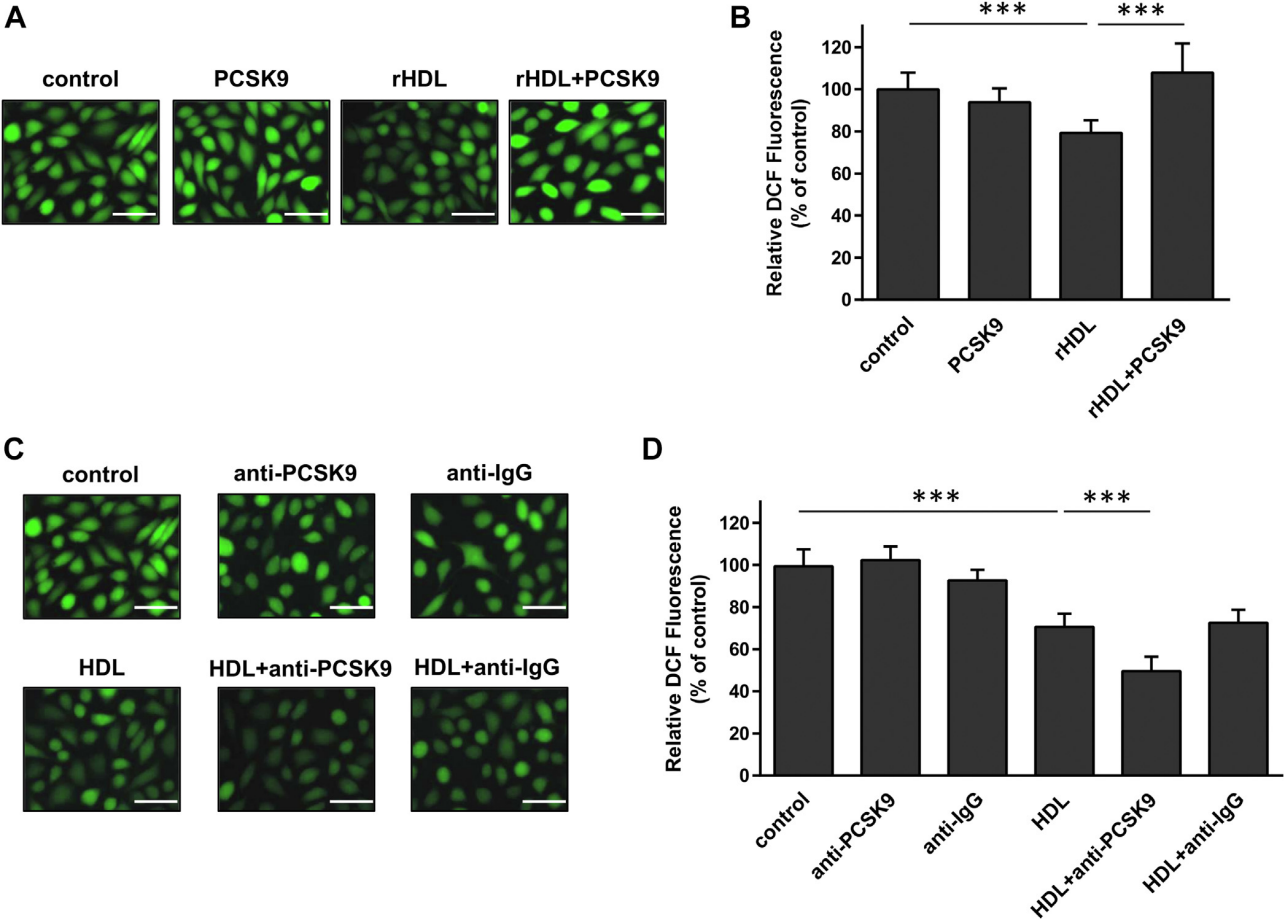
Effect of PCSK9 on the capacity of HDL to reduce ROS formation in endothelial cells. The formation of ROS by EA.hy926 cells treated with or without rHDL, in the absence or presence of PCSK9 (A and B), and with or without HDL (in apoB-depleted plasma), in the absence or presence of anti-human PCSK9 or anti-IgG (C and D), was measured following incubation of cells with DCFH-DA and detection of fluorescent DCF emitted from cells using a fluorescence microscope as described under “Materials and methods”. Representative microscopic views are shown (A and C). Scale bar = 100 μm. The same picture is used for control in panels (A) and (C) since the various treatments of cells were run in parallel. Images were scanned and quantified by ImageJ software. The relative DCF fluorescence is shown (B and D). Values are the means ± SD for three independent experiments performed in duplicate. ∗∗∗*P* < 0.0001. DCFH-DA, 2′,7′-dichlorodihydrofluorescein diacetate; DCF, 2′,7′-dichlorofluorescein; PCSK9, Proprotein Convertase Subtilisin/Kexin type 9; ROS, reactive-oxygen species; rHDL, reconstituted HDL.

The presence of PCSK9 in apoB-depleted plasma and at least partially in apoA-I–containing subpopulations of HDL prompted us to examine whether PCSK9 may have an effect on HDL functionality. Among the antiatherogenic functions of HDL is included its capacity to promote endothelial-protective effects, such as the protection from ROS and stimulation of cell migration (49, 50). Incubation of EA.hy926 endothelial cells with rHDL particles reduced the intracellular formation of ROS, as described before (42), while incubation of cells with rHDL in the presence of recombinant human PCSK9 failed to reduce ROS formation (Fig. 4A, B). Incubation of EA.hy926 cells with lipid-free PCSK9 had no effect on ROS formation, as compared to control cells. To provide evidence for the specificity of the preventive effect of PCSK9 on ROS formation by HDL, we treated EA.hy926 cells with apoB-depleted plasma, which contains HDL, alone or in combination with an anti-PCSK9 antibody. As shown in Fig. 4C, D, incubation of cells with HDL (in apoB-depleted plasma) reduced ROS formation, as expected, while coincubation of cells with HDL and the anti-PCSK9 antibody enhanced the reduction of ROS formation. Incubation of EA.hy926 cells with anti-PCSK9 in the absence of HDL (in apoB-depleted plasma) had no effect on ROS formation. In addition, incubation of cells with HDL (in apoB-depleted plasma) and a normal isotype IgG did not result in further reduction of ROS formation (Fig. 4C, D), demonstrating further the specificity of PCSK9 inhibition on HDL function.

**Fig. 5:**
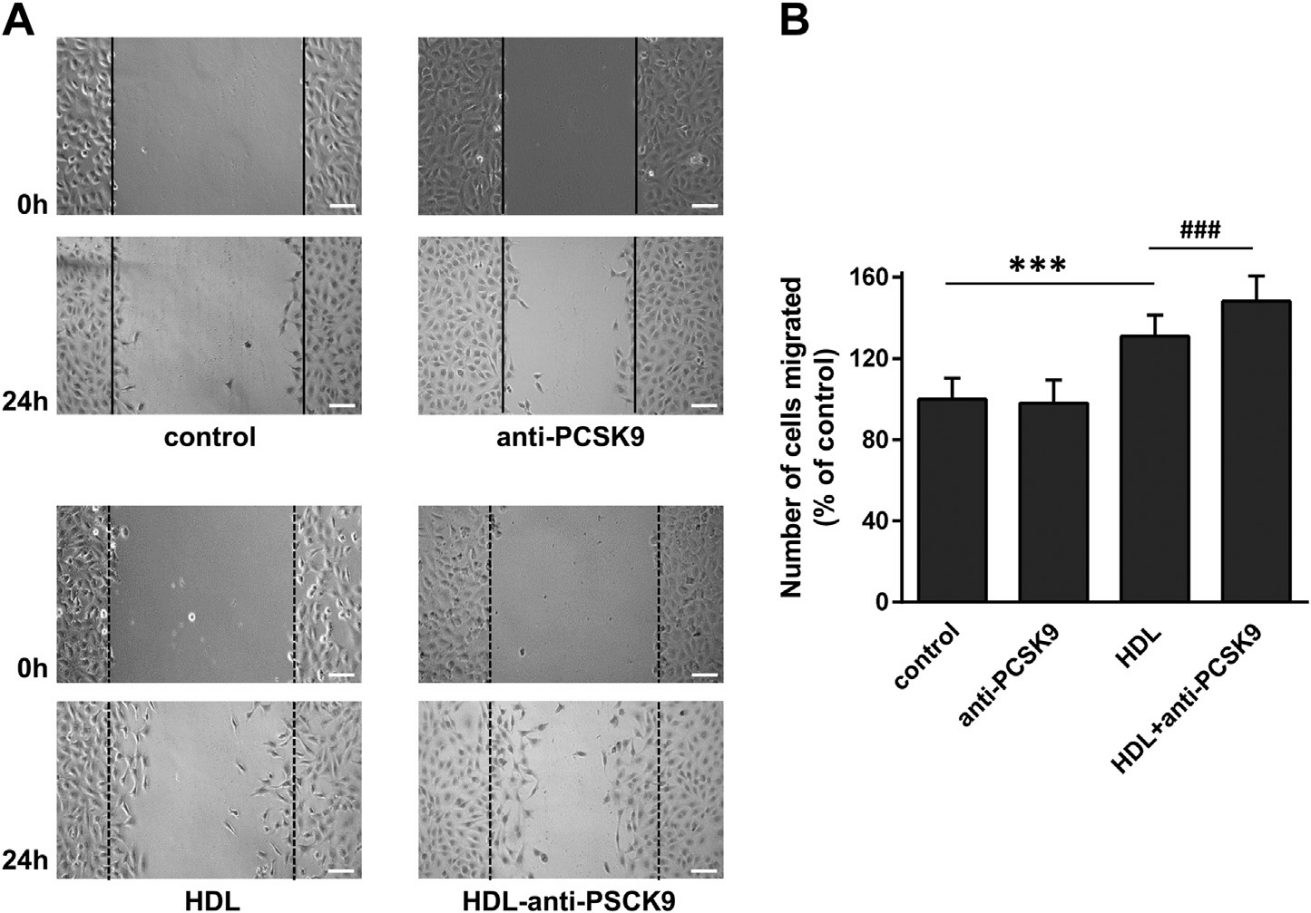
Effect of PCSK9 on the capacity of HDL to promote the migration of endothelial cells. EA.hy926 cells were wounded as described under “Materials and methods” and treated with or without HDL (in apoB-depleted plasma), in the absence or presence of anti-human PCSK9, for 24 h. Representative microscopic views at 0 h and 24 h are shown (A). Scale bar = 100 μm. Images were scanned and quantified by ImageJ software. The number of cells migrated after 24 h is shown (B). Values are the means ± SD for three independent experiments performed in duplicate. ∗∗∗*P* < 0.0001; ^###^*P* < 0.0005. PCSK9, Proprotein Convertase Subtilisin/Kexin type 9.

To examine further the effect of PCSK9 on the endothelial-protective effects of HDL, we studied the capacity of HDL (in apoB-depleted plasma) to stimulate endothelial cell migration in the absence or presence of an anti-PCSK9 antibody. When EA.hy926 cells were scratched and incubated with HDL (in apoB-depleted plasma) along with an anti-PCSK9 antibody, their migration was increased as compared to their migration when incubated with HDL alone (Fig. 5A, B). Collectively, our findings show that PCSK9 impairs the capacity of HDL to stimulate endothelial cells migration, as well as to protect endothelial cells from ROS production.

## Discussion

Circulating PCSK9 is primarily associated with LDL and Lpa particles (13, 20, 21) and has an important role in regulating LDL-cholesterol levels (3). However, here are contrasting results to date as to whether PCSK9 is also associated with HDL in human plasma (26, 27). Furthermore, contrasting results have been published regarding the correlation of PCSK9 with HDL-cholesterol levels in human plasma, since some studies showed a positive correlation (22, 23) whereas other studies failed to show this correlation (24, 25).

The present study shows that only a small fraction (6%) of PCSK9 in apoB-depleted plasma is associated with HDL particles. To study the possible association of PCSK9 with HDL particles in human plasma, we used plasma depleted of apoB-lipoproteins, which is prepared by much lower g forces as than ultracentrifugation methods. Our decision to avoid using ultracentrifugation methods to prepare HDL in the present study was based on previous results, showing that ultracentrifugation methods, which use high g forces, affect the proteomic composition of lipoproteins, including HDL (28, 47). Under these conditions, a dissociation of PCSK9 from LDL particles has been reported (20, 48). These findings are consistent with the present results showing that ultracentrifugation significantly reduces the PCSK9 content also of HDL, by primarily removing the mature PCSK9 thus leading to HDL particles containing only the furin-cleaved PCSK9 form. Our results demonstrated that approximately, the 32% of total PCSK9 in normolipidemic plasma remains in the supernatant after precipitation of apoB-lipoproteins and is consisted of the mature and the furin-cleaved forms. Importantly, only the 6% of this PCSK9 remaining in this supernatant is associated with HDL, while the majority exists in a free protein form. This HDL carries both forms of PCSK9.

HDL particles are heterogeneous in composition and structure, and it has been suggested that different subpopulations of HDL can exert different atheroprotective functions (51). In addition, several studies have examined the correlation of levels of HDL particles at various sizes with cardiovascular risk. α1 HDL levels in male participants in the Framingham Offspring Study have been found to display an inverse association, while α3 particle levels a positive association with the prevalence of coronary heart disease (52). Furthermore, an altered HDL subpopulation profile in coronary heart disease patients marked with low α1 and α2 particle levels and high α3 particle levels indicated an elevated risk for new events of cardiovascular disease (53). Other studies also showed that smaller HDL particles were found in women with cardiovascular disease compared to healthy subjects (54), while significantly smaller HDL particles, and specifically more HDL3 and less HDL2b particles, were found in patients with acute ischemic stroke (55). According to our results, obtained from the nondenaturing PAGE experiments, the L-HDL and I-HDL subfractions contain only the furin-cleaved PCSK9, whereas the S-HDL subfraction contains only the mature PCSK9 form. Thus, based on our results, we may suggest that the presence of mature active PCSK9 in S-HDL subfraction may contribute to the elevated cardiovascular risk associated with these HDL particles. Further studies in healthy subjects and cardiovascular disease patients are necessary to evaluate the role of HDL-associated PCSK9 forms in cardiovascular risk.

HDL and apoA-I, its main protein constituent, display several atheroprotective properties, including cholesterol efflux capacity, antioxidant, anti-inflammatory, and endothelial-protective properties (28). In a previous study, it was shown that PCSK9 inhibits the ABCA1-dependent cholesterol efflux from macrophages in the presence of apoA-I as acceptor (56). This inhibition, which was attributed to downregulation of *Abca1* gene and ABCA1 protein expression, suggested that PCSK9 can also have extrahepatic effects that may influence relevant steps in the pathogenesis of atherosclerosis (56). The possible influence though of PCSK9 on HDL functionality has not been studied.

In the current study, we present data on the role of PCSK9 on functions of HDL that are related to endothelium protection. HDL can promote the repair of the endothelium barrier by stimulating the migration of endothelial cells, while an additional vasoprotective function of HDL includes the inhibition of ROS production (57). Endothelial cell migration is involved in the early steps of repair of injured atherosclerotic vessels (58). Furthermore, migration of circulating endothelial progenitor cells to the injured endothelium was inversely associated with the occurrence of cardiovascular events (59), while the impairment of endothelial cell migration has been related to increased cardiovascular risk (42). ROS production in endothelial cells has been shown to induce the dimerization of endothelial nitric oxide synthase leading to decreased NO-induced vascular relaxation (60) and therefore inhibition of ROS production is important for the maintenance of endothelium integrity. Therefore, the observed effect of PCSK9 on reducing the capacity of HDL to promote migration of endothelial cells and to inhibit ROS formation may affect the functionality of HDL against atheroprotection, while the inactivation of PCSK9 in HDL may be desirable for improving the atheroprotective functions of HDL.

A limitation of the present study is that in the experiment with endothelial cells, we used apoB-depleted plasma containing the total plasma HDL as well as the plasma proteins including the free form of PCSK9 and not isolated HDL particles. Thus, from the results of present study, we cannot conclude as to whether the negative effect of PCSK9 in HDL endothelial-protective function is primarily attributed to PCSK9 associated with HDL or to the free PCSK9 form in apoB-depleted plasma. In any case though, our studies show that PCSK9 in apoB-depleted plasma does affect the atheroprotective properties of HDL indicating a novel role of PCSK9 in atherosclerosis. Another limitation of the present study is that we were not able to calculate the concentration of PCSK9 in each HDL subfraction (expressed per HDL-cholesterol or HDL mass) due to the low yield of the method used to excise the three subfractions. However, our study clearly demonstrates that PCSK9 is present in all three subfractions (L-HDL, I-HDL, S-HDL) and the largest proportion is migrated at the region where the small S-HDL particles are migrated.

In conclusion, we show for the first time that the mature active PCSK9 form is associated with S-HDL whereas the furin-cleaved PCSK9 form is associated with L-HDL and I-HDL subfractions. The levels of active and cleaved PCSK9 forms in HDL should be further investigated for their potential use as biomarkers for cardiovascular disease. In addition, the observed effect of PCSK9 in the impairment of HDL atheroprotective functions highlights further the importance of PCSK9 inhibition against atherosclerosis and risk of cardiovascular disease.

## Data availability

All data is located at Atherothrombosis Research Center and will be available upon request from the corresponding author Professor Alexandros D. Tselepis (atselep@uoi.gr).

## Conflict of interest

The authors declare that they have no conflicts of interest with the contents of this article.
